# Antimicrobial susceptibility testing reveals reduced susceptibility to azithromycin and other antibiotics in *Legionella pneumophila* serogroup 1 isolates from Portugal

**DOI:** 10.1007/s10096-024-04789-9

**Published:** 2024-05-02

**Authors:** Corrado Minetti, Rachael Barton, Caitlin Farley, Owen Brad Spiller, Raquel Rodrigues, Paulo Gonçalves

**Affiliations:** 1 Department of Infectious Diseases, National Institute of Health Doctor Ricardo Jorge (INSA), Lisbon, Portugal; 2 ECDC Fellowship Programme, Public Health Microbiology Path (EUPHEM), European Centre for Disease Prevention and Control (ECDC), Stockholm, Sweden; 3 Department of Medical Microbiology, Division of Infection and Immunity, School of Medicine, Cardiff University, Cardiff, United Kingdom; 4 Water Microbiology Laboratory, National Institute of Health Doctor Ricardo Jorge (INSA), Lisbon, Portugal; 5 National Reference Laboratory for Legionella, National Institute of Health Doctor Ricardo Jorge (INSA), Lisbon, Portugal

**Keywords:** Legionnaires' disease, Legionella pneumophila, Antimicrobial susceptibility, Antibiotic resistance, Surveillance, Public health, Portugal

## Abstract

**Backgroud:**

Although not fully investigated, studies show that *Legionella pneumophila* can develop antibiotic resistance. As there is limited data available for Portugal, we determined the antibiotic susceptibility profile of Portuguese *L. pneumophila* serogroup 1 (LpnSg1) isolates against antibiotics used in the clinical practice in Portugal.

**Methods:**

Minimum inhibitory concentrations (MICs) were determined for *LpnSg1* clinical (*n* = 100) and related environmental (*n* = 7) isolates, collected between 2006–2022 in the context of the National Legionnaire´s Disease Surveillance Programme, against azithromycin, clarithromycin, erythromycin, levofloxacin, ciprofloxacin, moxifloxacin, rifampicin, doxycycline, tigecycline, and amoxicillin/clavulanic acid, using three different assays. Isolates were also PCR-screened for the presence of the *lpeAB* gene.

**Results:**

Twelve isolates had azithromycin MICs above the EUCAST tentative highest WT MIC, 9 of which were *lpeAB* negative; for erythromycin and clarithromycin, all isolates tested within the susceptible range. The number of isolates with MICs above the tentative highest WT MIC for the remaining antibiotics was: ciprofloxacin: 7; levofloxacin: 17; moxifloxacin: 8; rifampicin: 11; doxycycline: 82; tigecycline: 4. EUCAST breakpoints are not available for amoxicillin/clavulanic acid. We estimated the ECOFFs and one isolate had a MIC eightfold higher than the E-test ECOFF. Additionally, a clinical isolate generated three colonies growing on the E-test inhibition zone that resulted in MICs fourfold higher than for the parental isolate.

**Conclusions:**

We report, for the first time, elevated MICs against first-line and other antibiotics (including azithromycin, fluoroquinolones and amoxicillin/clavulanic acid commonly used to treat pneumonia patients in Portugal) in Portuguese *L. pneumophila* strains. Results point towards decreased susceptibility in circulating strains, justifying further investigation.

**Supplementary Information:**

The online version contains supplementary material available at 10.1007/s10096-024-04789-9.

## Introduction

The Gram-negative bacillus *Legionella pneumophila* is clinically associated with Legionnaires´ Disease (LD), a severe form of community-acquired pneumonia (CAP) [1]. If untreated, the case fatality rate of LD can be up to 10% [2]. *L. pneumophila* serogroup 1 (sg1) is responsible for the majority of cases worldwide, including in Europe [2]. Although LD is relatively sporadic in Europe, with high heterogeneity in reporting between countries, notification rates have been increasing from 1.4 to 2.2 cases/100000 population between 2016 and 2021 [3], and large outbreaks have also been reported in recent years [4–6].

Since *Legionella* replicates intracellularly, the choice of therapeutics for LD is limited to antimicrobials which can penetrate cells such as macrolides or fluoroquinolones [7]. Azithromycin, levofloxacin, or moxifloxacin are recommended as first-line treatment of LD [8], but β-Lactams such as amoxicillin are also frequently used as first option to treat patients with CAP [9]. In Portugal, the recommended therapeutics for previously healthy CAP patients include amoxicillin as first option, with azithromycin, clarithromycin, or doxycycline as alternatives [10]. In patients with comorbidities or with recent antibiotic therapy, the recommendations are to administer amoxicillin in combination with azithromycin, clarithromycin, or doxycycline, and levofloxacin or moxifloxacin as alternatives [10].

Although antibiotic resistance in *Legionella* has not yet been a subject for major concern, it has indeed been observed. Ciprofloxacin-resistant bacteria have been isolated from patients undergoing treatment [11, 12]. A study including clinical isolates found an association between reduced susceptibility to erythromycin and azithromycin and the presence of *lpeAB* genes (coding for an efflux pump involved in macrolide resistance) [13, 14]. In another study, mutations in the promoter region of these genes were found in in vitro selected resistant strains after exposure to azithromycin, and a more recent study showed that environmental isolates highly resistant to macrolides carried mutations in the 23S rRNA gene [15, 16].

In vitro antibiotic susceptibility testing (AST) is crucial to determine the minimum inhibitory concentration (MIC) of the drugs and to assess whether bacteria are showing signs of resistance. However, there is no gold standard method recommended for *Legionella*. Available methods include agar dilution, broth microdilution (BMD) and gradient strip testing on buffered charcoal-containing yeast extract (BCYE) agar [7]. BMD is usually considered the most reliable method for clinically relevant bacteria, but it is time-consuming due to the slow growth rate and complex medium requirements of *Legionella*. Gradient strips are more widely used due to their ease of use, although they tend to produce MIC values that are higher than those returned by BMD in consequence of the use of charcoal in the medium. Recently a new method using a solid charcoal-free medium (LASARUS) [17] has shown to produce results more in line with those of BMD, but it still needs further validation. The European Committee on Antimicrobial Susceptibility Testing (EUCAST) has produced guidelines for the interpretation of MICs in *Legionella* using either the BMD or the gradient strip methods [18], based on the highest MIC observed in wild type (WT) isolates from published studies. These values are used as a threshold for submitting the isolates to a reference laboratory for further testing, but there is no universal agreement on epidemiological cut-off (ECOFF) values to discriminate between wild-type and potentially resistant strains partly due to the different MIC values returned by different in vitro methods [19]. Additionally, there are currently no clinical breakpoints available for *Legionella* to define whether an infection is likely to be treatable or not in a patient [20]. Given the increasing trend in LD notifications and its severity, there is a need for a more extensive antimicrobial susceptibility screening of both clinical and environmental *L. pneumophila* strains to have a clearer picture of the situation at the European level. Additionally, comparing different in vitro AST methods is pivotal to help refining and standardising the current methods and guidelines for the determination and interpretation of MICs and cut-off values for *Legionella*.

There is little information on the antibiotic susceptibility profile of *L. pneumophila* strains circulating in Portugal. A study from 1997 including both clinical and environmental isolates did not find evidence of reduced susceptibility [21]. However, a more recent study involving strains isolated from water samples found evidence of potential resistance to levofloxacin [22].

Our study aimed to determine the antibiotic susceptibility of *L. pneumophila* serogroup 1 clinical and environmental isolates collected in Portugal between 2006 and February 2022 to ten antibiotics used in the clinical practice. Three AST methods were used (gradient strip, BMD, and LASARUS agar medium). Additionally, we determined the prevalence of the *lpeAB* gene.

## Materials and methods

### Bacterial strains

A total of 107 *L. pneumophila* sg1 isolates were tested: 100 clinical (of which 72 from sporadic LD cases, and 28 from 11 confirmed outbreaks) and 7 environmental (associated with 7 of the 11 confirmed outbreaks). The isolates were collected between 2006 and February 2022 in the context of the National Legionnaires’ Disease Surveillance Programme and stored at the National Reference Laboratory for *Legionella* of the National Institute of Health Doutor Ricardo Jorge (NRL/INSA) in Lisboa, Portugal. Available metadata and the sequence type (ST) of the isolates, determined with the standard 7-alleles sequence-type based protocol [23], are reported in the Supplementary file.

### Antimicrobial susceptibility testing

Isolates stored at < -70 °C were inoculated on buffered charcoal-containing yeast extract medium supplemented with α-ketoglutarate (BCYE-α) and incubated at 36 ± 2 °C in a humid chamber for 48–72 h before testing. The fully susceptible *L. pneumophila* subsp. *pneumophila* Philadelphia-1 strain (ATCC 33152) was inoculated in the same way and used as a reference strain. Bacteria were tested for the following ten antibiotics: azithromycin, clarithromycin, erythromycin, levofloxacin, ciprofloxacin, moxifloxacin, rifampicin, doxycycline, tigecycline, and amoxicillin/clavulanic acid (2/1). AST was performed by three methods. The gradient strip method was performed at the NRL/INSA following the recommendations of EUCAST [18] and using E-test® gradient strips (bioMérieux SA, France) according to the manufacturer’ instructions [24]. All isolates were also shipped to the Department of Medical Microbiology, Cardiff University School of Medicine, United Kingdom, where they were tested for the same antibiotics using the BMD and LASARUS agar methods as previously described [17]. Broth and agar dilution MICs were determined in duplicate (biological repeat, separate day) for each isolate. While an uncommon occurrence, if the MICs for each isolate differed by more than one dilution a third replicate was performed to remove outlier results. MICs were read as the lowest antimicrobial concentration inhibiting growth. Additionally, DNA was extracted from all the isolates and used for the PCR amplification of the *lpeAB* gene as previously described [13].

### Data analysis

MIC values were used to classify the isolates as susceptible (MIC below or equal to the tentative highest WT MIC) or with reduced susceptibility (MIC above the tentative highest WT MIC) according to the EUCAST guidelines [18]. MIC values were used to calculate the minimum concentration at which 50% (MIC_50_) and 90% (MIC_90_) of the isolates are inhibited, respectively, and the MIC range. Additionally, ECOFFs (95%) were calculated using the ECOFFinder program (version 2.1) [25, 26]. MIC and ECOFF (if available) values were extrapolated from other representative published studies for comparison. Data were tabulated and graphs were constructed using Microsoft Excel.

## Results

**Fig. 1:**
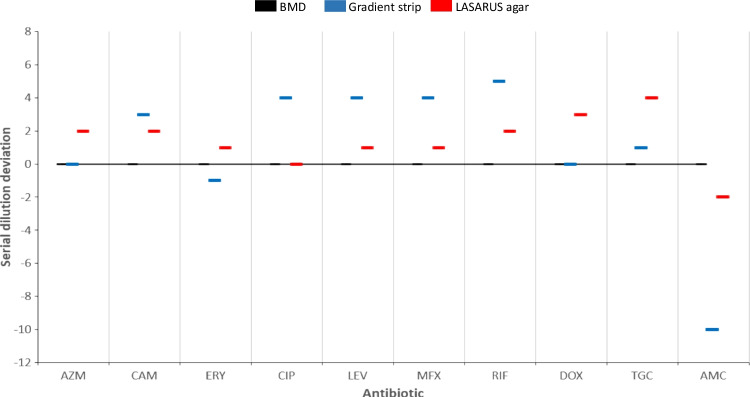
Modal averages of MIC values by gradient strip and LASARUS agar expressed as serial dilution deviation from the BMD gold standard (black line). AZM = azithromycin; CAM = clarithromycin; ERY = erythromycin; CIP = ciprofloxacin; LEV = levofloxacin; MFX = moxifloxacin; RIF = rifampicin; DOX = doxycycline; TGC = tigecycline; AMC = amoxicillin/clavulanic acid

The MIC distribution for the tested antibiotics of the 107 isolates is shown in Table 1, while a summary of the number of isolates showing reduced susceptibility to the antibiotics is shown in Table 2. The MIC range, MIC_50_, MIC_90_ and ECOFF values are reported in Table 3 (the ECOFFinder fitted curves are available as a supplementary Excel file). The latter table also reports the values from representative studies as a comparison. The mode of deviation of the MIC values obtained by gradient strip and LASARUS compared to the BMD gold standard are shown in Fig. 1.

**Table 1:**
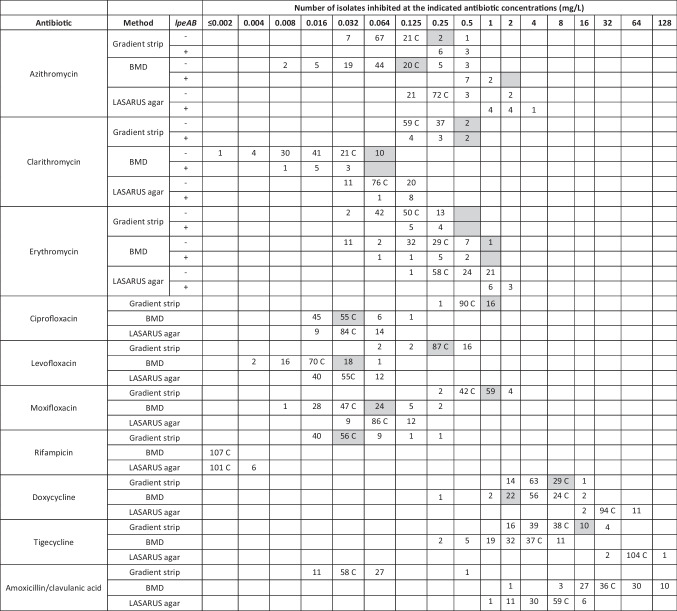
MIC distribution of the *L. pneumophila* serogroup 1 isolates from Portugal (*n* = 107)

Tentative EUCAST highest WT MIC values are highlighted in grey. The reference control strain is indicated as C

**Table 2:** Summary of the number and percentage of isolates with MIC above the EUCAST highest WT MIC and the ECOFF values calculated in this study in the *L. pneumophila* serogroup 1 isolates from Portugal (*n* = 107)

		No. isolates (%)			
Antibiotic	Criterium	By gradient strip	By BMD	By both	Total
Azithromycin	MIC > EUCAST highest WT	4 (3.7)	8 (7.5)	0 (0)	**12 (11.2)**
	MIC > ECOFF from this study#	1 (10.2)	3 (3.1)	0 (0)	**4 (4.1)**
Clarithromycin	MIC > ECOFF from this study	6 (5.6)	0 (0)	0 (0)	**6 (5.6)**
Ciprofloxacin	MIC > EUCAST highest WT	0 (0)	7 (6.5)	0 (0)	**7 (6.5)**
	MIC > ECOFF from this study	0 (0)	7 (6.5)	0 (0)	**7 (6.5)**
Levofloxacin	MIC > EUCAST highest WT	16 (14.9)	1 (0.9)	0 (0)	**17 (15.9)**
	MIC > ECOFF from this study	0 (0)	0 (0)	0 (0)	**0 (0)**
Moxifloxacin	MIC > EUCAST highest WT	4 (3.7)	7 (6.5)	3 (2.8)	**8 (7.5)**
	MIC > ECOFF from this study	4 (3.7)	7 (6.5)	3 (2.8)	**8 (7.5)**
Rifampicin	MIC > EUCAST highest WT	11 (10.3)	0 (0)	0 (0)	**11 (10.3)**
	MIC > ECOFF from this study	2 (1.8)	0 (0)	0 (0)	**2 (1.8)**
Doxycycline	MIC > EUCAST highest WT	0 (0)	81 (75.7)	1 (0.9)	**82 (76.6)**
	MIC > ECOFF from this study	0 (0)	2 (1.8)	0 (0)	**2 (1.8)**
Tigecycline	MIC > EUCAST highest WT	4 (3.7)	0 (0)	0 (0)	**4 (3.7)**
	MIC > ECOFF from this study	4 (3.7)	0 (0)	0 (0)	**4 (3.7)**
Amoxicillin/clavulanic acid	MIC > ECOFF from this study	1 (0.9)	0 (0)	0 (0)	**1 (0.9)**

^#^excluding the *lpeAB* + isolates

**Table 3:** Minimum inhibitory concentration ranges, MIC_50_, MIC_90_ and ECOFF values of the *L. pneumophila* serogroup 1 isolates from Portugal (*n* = 107) compared to other representative studies*

Antibiotic	Method	Source	MIC_50_ (mg/L)	MIC_90_ (mg/L)	MIC range (mg/L)	ECOFF (mg/L)
Azithromycin	Gradient strip	**This study**	**0.064**	**0.25**	**0.032–0.5**	**0.250**
		UK [17]	0.064	0.128	0.032–0.25	−
		Germany [27]	0.125	1	0.032–1	−
		Norway [14]	0.125	0.5	0.032–1	−
		Italy [28]	0.19	0.5	−	−
		Israel [29]	0.38	0.75	0.032–1	2
	BMD	**This study**	**0.064**	**0.5**	**0.008–1**	**0.250**
		Portugal [22]	0.25	0.5	0.064–0.5	2
		UK [17]	0.032	0.064	0.008–0.25	−
		France [13]	0.064	0.5	0.015–2	2
	LASARUS agar	**This study**	**0.25**	**0.5**	**0.125–4**	**1**
		UK [17]	0.032	0.064	0.008–0.064	−
Clarithromycin	Gradient strip	**This study**	**0.125**	**0.25**	**0.125–0.5**	**0.25**
		Germany [27]	0.25	0.5	0.032–0.5	−
		Italy [28]	0.032	0.125	−	−
		Israel [29]	0.064	0.25	0.025–0.5	0.5
	BMD	**This study**	**0.016**	**0.032**	**0.002–0.064**	**0.064**
		Portugal [22]	0.064	0.064	0.032–0.5	0.5
		France [13]	0.032	0.032	0.004–0.064	0.064
	LASARUS agar	**This study**	**0.064**	**0.125**	**0.032–0.125**	**0.125**
Erythromycin	Gradient strip	**This study**	**0.064**	**0.25**	**0.032–0.25**	**0.25**
		Portugal [21]	0.38	2	0.125–8	−
		UK [9]	0.25	0.5	0.064–1	−
		Germany [27]	0.25	0.5	0.064–1	−
		Norway [14]	0.25	0.5	0.064–1	−
		Italy [28]	0.094	0.19	−	−
		Israel [29]	0.094	0.5	0.023–1	0.5
	BMD	**This study**	**0.125**	**0.25**	**0.032–1**	**0.5**
		France [13]	0.125	0.5	0.032–1	1
	LASARUS agar	**This study**	**0.25**	**1**	**0.125–2**	**0.5**
Ciprofloxacin	Gradient strip	**This study**	**0.5**	**1**	**0.25–1**	**1**
		Portugal [21]	0.5	0.75	0.25–1	−
		Germany [27]	0.5	0.5	0.25–1	−
		Norway [14]	0.5	0.5	0.25–1	−
		Italy [28]	0.19	0.38	−	−
		Israel [29]	0.75	1.5	0.019–2	4
	BMD	**This study**	**0.032**	**0.032**	**0.016–0.125**	**0.032**
		Portugal [22]	0.032	0.125	0.032–32	0.25
		UK [9]	0.015	0.032	0.004–0.25	−
		France [13]	0.016	0.032	0.008–0.064	0.064
	LASARUS agar	**This study**	**0.032**	**0.032**	**0.016–0.064**	**0.064**
Levofloxacin	Gradient strip	**This study**	**0.25**	**0.5**	**0.064–0.5**	**0.5**
		UK [17]	0.064	0.128	0.064–0.5	−
		Germany [27]	0.25	0.5	0.032–0.5	−
		Norway [14]	0.25	0.25	0.125–25	−
		Italy [28]	0.064	0.094	−	−
		Israel [29]	0.075	1	0.023–1.5	1
	BMD	**This study**	**0.016**	**0.032**	**0.004–0.064**	**0.032**
		Portugal [22]	0.032	0.032	0.016–16	0.25
		UK [17]	0.032	0.032	0.008–0.064	−
		UK [9]	0.064	0.125	0.03–0.25	−
		France [13]	0.016	0.032	0.004–0.032	0.032
	LASARUS agar	**This study**	**0.032**	**0.064**	**0.016–0.064**	**0.064**
		UK [17]	0.032	0.032	0.008–0.032	−
Moxifloxacin	Gradient strip	**This study**	**1**	**1**	**0.25–2**	**1**
		Germany [27]	0.5	1	0.25–1	−
		Norway [14]	0.5	1	0.25–1	−
		Italy [28]	0.25	0.25	−	−
		Israel [29]	0.5	1	0.032–1.5	4
	BMD	**This study**	**0.032**	**0.064**	**0.008–0.25**	**0.064**
		UK [9]	0.125	0.125	0.032–0.25	−
		France [13]	0.032	0.032	0.008–0.064	0.064
	LASARUS agar	**This study**	**0.064**	**0.125**	**0.032–0.125**	**0.125**
Rifampicin	Gradient strip	**This study**	**0.032**	**0.032**	**0.016–0.25**	**0.064**
		Portugal [21]	0.023	0.094	0.016–0.5	−
		UK [17]	0.016	0.032	0.008–0.125	−
		Germany [27]	0.016	0.032	0.008–0.032	−
		Norway [14]	0.016	0.032	0.004–0.032	−
		Italy [28]	0.016	0.016	−	−
		Israel [29]	0.023	0.5	0.003–1	0.064
	BMD	**This study**	** < 0.002**	** < 0.002**	** < 0.002**	**0.002**
		UK [17]	0.004	0.008	0.001–0.008	−
		UK [9]	0.0001	0.0001	0.0001	−
		France [13]	0.0005	0.0005	0.00012–0.001	0.001
	LASARUS agar	**This study**	**0.002**	**0.002**	**0.001–0.004**	**0.004**
		UK [17]	0.004	0.008	0.0005–0.008	−
Doxycycline	Gradient strip	**This study**	**4**	**8**	**2–16**	**8**
		Portugal [21]	2	3	1–6	−
		UK [17]	2	4	1–8	−
		Germany [27]	1	2	0.5–4	−
		Italy [28]	1.5	3	−	−
		Israel [29]	0.032	0.5	0.023–0.5	0.5
	BMD	**This study**	**4**	**8**	**0.25–16**	**8**
		Portugal [22]	4	16	2–16	64
		UK [17]	16	32	2–32	−
		France [13]	1	2	0.12–2	2
	LASARUS agar	**This study**	**32**	**32**	**2–64**	**64**
		UK [17]	16	32	2–32	−
Tigecycline	Gradient strip	**This study**	**4**	**16**	**2–32**	**16**
		Italy [28]	1.5	4	−	−
		Israel [29]	0.5	1.5	0.023–2	0.5
	BMD	**This study**	**2**	**8**	**0.25–8**	**8**
	LASARUS agar	**This study**	**64**	**64**	**32–128**	**128**
Amoxicillin/clavulanic acid	Gradient strip	**This study**	**0.032**	**0.064**	**0.016–0.5**	**0.064**
		Norway [14]	< 0.016	< 0.016	< 0.016	−
	BMD	**This study**	**8**	**8**	**1–128**	**128**
	LASARUS agar	**This study**	**8**	**8**	**1–16**	**16**

MIC_50_/MIC_90_ = lowest antibiotic concentration at which 50% and 90% of isolates were inhibited, respectively; BMD = broth microdilution; LASARUS = charcoal-free solid medium. Country, reference, number and type of isolates tested by study: Portugal [22] 8 environmental, all sg1; Portugal [21] 16 clinical (15 sg1, 1 sg14) + 14 environmental (6 sg1, 1 sg10, 7 other *Legionella* spp.); UK [17] 27 clinical + 13 environmental, all sg1; UK [9] 71 clinical, all sg1; Germany [27] 100 clinical, all sg1; Norway [14] 55 clinical (54 sg1, 1 sg4) + 67 environmental (65 sg1, 2 sg5); Italy [28] sg1 environmental samples (number not available); Israel [29] 12 clinical all sg1 + 93 environmental (92 sg1, 1 sg3); France [13] 109 clinical, all sg1. *This table is limited to a comparison to ESCMID-participating sites and should not be considered an exhaustive list for global studies

### lpeAB gene screening

A total of 9 (8.4%) isolates were found carrying the *lpeAB* gene.

### Macrolides

In total, 12 isolates (11.2%) had azithromycin MICs above the tentative highest WT MIC: 4 (3.7%) by gradient strip (1 *lpeAB*-negative and 3 *lpeAB*-positive) and 8 (7.5%) by BMD (threshold 0.125 mg/L, all *lpeAB*-negative). Overall the BMD MIC for azithromycin was statistically higher for *lpeAB*-positive isolates compared to the rest (0.1032 ± 0.0909 vs 0.5833 ± 0.2500 mg/L; p < 0.001 by students t-test). For erythromycin and clarithromycin, all isolates tested within the susceptible range by both the gradient test and BMD; however, the BMD MICs for these two macrolides were not sufficiently elevated to achieve statistical difference comparing *lpeAB*-positive and *lpeAB*-negative isolates. Estimated ECOFF values using ECOFFinder in our study differed from the highest WT MIC values published by EUCAST [20]. Our values were two-fold lower with the gradient strip for clarithromycin and erythromycin and with the BMD for erythromycin, while the ECOFF with the BMD method for azithromycin was two-fold higher than the EUCAST value.

### Fluoroquinolones

Isolates with MICs above the tentative highest WT MIC for the tested fluoroquinolones were found: 7 (6.5%) for ciprofloxacin (all BMD, MIC > 0.032 mg/L), 17 (15.9%) for levofloxacin (16 for gradient strip MIC > 0.25; 1 for BMD MIC > 0.032 mg/L), and 11 (7.5%) for moxifloxacin (4 for BMD MIC > 1; 7 for BMD MIC > 0.064). By considering either gradient strip or BMD results, a total of 4 (3.7%) isolates had reduced susceptibility for all three fluoroquinolones and an additional 3 (2.8%) had reduced susceptibility for both ciprofloxacin and moxifloxacin. The ECOFF value for levofloxacin using ECOFF finder obtained with gradient strip was two-fold higher than the highest WT MIC values published by EUCAST [18], while the BMD ECOFFs corresponded to putative susceptibility thresholds suggested by EUCAST.

### Rifampicin

A total of 11 (10.3%) isolates had a MIC above the tentative highest WT MIC for rifampicin (only for by gradient strip MIC > 0.032 mg/L). Our BMD MICs corresponded to values indicated by EUCAST [18], while the ECOFF determined by ECOFF finder for the gradient strip was two-fold higher than EUCAST recommends.

### Tetracyclines

Overall, 82 isolates (76.6%) had doxycycline MICs above the tentative highest WT MIC (1 for gradient strip MIC > 8 mg/L, the rest were BMD MIC > 2 mg/L), and 4 of these (3.7% out of the total tested) also had reduced susceptibility for tigecycline (gradient strip MIC > 16; no value available for BMD to assess). The ECOFF from ECOFF finder for both antibiotics for gradient strip corresponded to highest WT MIC from EUCAST, while the ECOFF for doxycycline for BMD was four-fold higher than identified by EUCAST.

### Amoxicillin/clavulanic acid

Tentative EUCAST highest WT MIC values for this antibiotic are not available for *Legionella*, but considering the gradient strip test and the relative estimated ECOFF (0.064 mg/L), one isolate (0.9%) had a MIC above it. During testing of one of the clinical isolates (E206-0) with the gradient strip test, three colonies were observed growing within the inhibition halo. The colonies were then re-isolated and tested separately. All three colonies (labelled E206-1 to -3) returned a MIC two- to four-fold higher than that observed for the initial isolate by both the gradient strip and BMD tests, and sixteen-fold higher with the LASARUS agar test. The three colonies also showed elevated MICs compared to the initial isolate for the following antibiotics: ciprofloxacin (four-fold higher by both BMD and LASARUS agar), levofloxacin (two-fold higher in one colony by BMD and in all three by LASARUS agar; four-fold higher in two colonies by BMD only), and both azithromycin and clarithromycin (two-fold higher by BMD only). The three colonies also showed a two-fold lower MIC for tigecycline compared to the initial isolate (by BMD only), and one colony had a two-fold lower MIC for azithromycin.

### Reduced susceptibility to multiple antibiotics

By taking into account either gradient strip or BMD results, 16 isolates (14.9%) had reduced susceptibility to both levofloxacin and doxycycline, 12 (11.2%) to azithromycin and doxycycline, and 10 (9.3%) to rifampicin and doxycycline. Other occurrences included reduced susceptibility to ciprofloxacin/moxifloxacin and doxycycline (6 isolates each, 5.6%), levofloxacin and rifampicin/tigecycline (4 isolates each, 3.7%), rifampicin and tigecycline (2 isolates, 1.9%), and azithromycin and rifampicin (1 isolate, 0.9%).

### Comparison of MICs between the three AST methods

Compared to the BMD gold standard (Fig. 1), the gradient strip returned significantly elevated thresholds of inhibition for clarithromycin (three serial dilutions), all fluoroquinolones (four dilutions), and rifampicin (five dilutions). The gradient strip also showed a significantly reduced threshold (down to ten dilutions) compared to BMD for amoxicillin/clavulanic acid. The LASARUS agar method returned MIC values which were more comparable to those by BMD, with all thresholds of inhibition showing a deviation of plus/minus two dilution factors except for tigecycline (four dilutions). The deviations of gradient strip and LASARUS results from the BMD are reflected also by MIC ranges, MIC_50_, MIC_90_ and ECOFF values (Table 2).

## Discussion

Our study generated the first data on the antibiotic susceptibility profile of *L. pneumophila* serogroup 1 isolated from Portuguese LD patients since 1997 [21]. Compared to a recent Portuguese study using only broth microdilution [22], we used in addition another method recommended by EUCAST (gradient strip) and the recently proposed described LASARUS agar dilution method [17].

Overall, the MIC values and ranges of our isolates were comparable (with minor variations) to those reported in other studies using the gradient strip and/or the BMD methods. Rifampicin was the most effective antibiotic, while doxycycline and tigecycline were the least effective. These results are in accordance to those reported by other authors [13, 17, 27, 28, 30]. To classify the isolates as susceptible or (potentially) resistant we used the MIC thresholds recommended by EUCAST for referring isolates to reference laboratories as putatively resistant [18]. It is important to note that these reference values are based on literature review and differ between the gradient strip and BMD approaches. While an isolate can have the same MIC by both methods for some antibiotics, it can be differentially classified as susceptible because the literature for one method shows a higher average range. Nevertheless, we found evidence of reduced susceptibility to various antibiotics, including to first-line compounds.

We found twelve isolates with reduced susceptibility to azithromycin. Interestingly, nine of these isolates were not carrying the *lpeAB* gene which is known to confer resistance to macrolides [13, 14, 16], and we did not have any information regarding treatment of the patient. As expected, most of (but not all) the isolates carrying this gene had the highest MICs. The lack of this gene in isolates with high azithromycin MIC is not unexpected and it has been previously reported [17, 31, 32], including in Portuguese environmental isolates [22]. The presence of the *lpeAB* gene did not seem to impact the MIC values for clarithromycin and erythromycin. This possible selective resistance for azithromycin, compared to the other macrolides, has also been previously observed in a study of 1464 environmental *L. pneumophila* from China [32]. These results suggest that other resistance/reduced susceptibility mechanisms might be involved. We did not perform any sequencing on these isolates to investigate the underlying molecular determinants (e.g. to determine mutations in 23S rRNA or the L4/L22 ribosome accessory proteins), as it was outside of the scope of this study and, therefore, the underlying mechanism for this phenotype remains unknown. However, mutations in the 23S rRNA of *L. pneumophila* sg1 generated by repeated in vitro challenge for resistance to macrolides showed MIC values well above 16 mg/L by BMD, which is much higher than we observed [13, 18]. Although none of our isolates had MIC values comparable to these, since azithromycin is expected to be one of the most frequent antibiotic administered to LD patients following standardised treatment guidelines, it will be important to continue to assess and monitor potential azithromycin resistance phenomena in Portugal. Although isolates did not show any reduced susceptibility for the other two macrolides tested, all the isolates with the *lpeAB* gene had MICs at the higher end of the range for erythromycin.

Some isolates also showed reduced susceptibility to fluoroquinolones, including four with MIC values above the breakpoints for all the antibiotics tested. However, while of particular interest phenotypically, the sequence of the *gyrA* and *parC* genes in E206-1, -2 and -3, did not show any alteration in the quinolone-resistance determining region relative to the E206-0 parent strain (data not shown), to explain the elevation in ciprofloxacin MIC. Two isolates from our sample additionally had a BMD MIC of 0.250 mg/L for moxifloxacin. Previous studies have reported a ciprofloxacin-resistant *L. pneumophila* strain isolated from a patient, showing a MIC of 2 mg/L by gradient strip [11], and in vitro selected strains showing MIC values above 0.125 mg/L by BMD for either levofloxacin or moxifloxacin [13, 18]. However, the underlying mechanisms for these observations remain so far unknown.

We found eleven isolates with reduced susceptibility to rifampicin (including two with a MIC four- and eight-fold higher than the ECOFF, respectively) by the gradient strip method. Using the same approach, a previous study reported isolates with MIC values up to 4 mg/L [28].

The majority (more than 70%) of our isolates showed very high inhibition thresholds for doxycycline by BMD (above the EUCAST reference thresholds for submission to reference laboratories as putatively resistant), although similar MIC values have been reported in UK [17] and Portuguese [22] environmental isolates before. Whether these values reflect naturally occurring variation in susceptibility, or the presence of resistance, requires further testing and analysis.

One of the most interesting results of our study came from the amoxicillin/clavulanic acid testing. No ECOFFs are available for this antibiotic combination in *Legionella*, and to the best of our knowledge only one study assessed the susceptibility of these bacteria to it [14]. Compared to the gradient strip results from that study (all isolates had a MIC < 0.016 mg/L) [14], 80% of our isolates had MIC values or 0.032 mg/L and above. We also managed to isolate three colonies showing signs of resistance to amoxicillin/clavulanic acid, as confirmed by a significantly increased threshold of inhibition compared to the parent isolate, not only by gradient strip but also by BMD. These three isolates underwent whole-genome sequencing and were confirmed to be the same as the parental isolate (National Reference Laboratory for *Legionella*, *pers. comm.*). Further analysis is underway to determine the potential molecular mechanism behind the phenotype. The finding that *L. pneumophila* can develop resistance to this antibiotic is very relevant for patient management and public health. β-Lactam antibiotics such as amoxicillin can be frequently used to treat patients with CAP [9], and this drug was the second most frequently reported as treatment for CAP patients according to surveillance data collected non-systematically at the National Reference Laboratory relative to the period of this study. However, it is important to note that β-Lactams would only be effective against extracellular *Legionella* and are unlikely to resolve the infection on their own.

We also estimated the epidemiological cut-off (ECOFF) values specifically in our sample to compare them to the EUCAST tentative highest WT MICs. While the ECOFFs obtained with BMD largely overlapped with the EUCAST threshold values (apart from being lower for erythromycin, and higher for doxycycline), more discrepancies were observed for values obtained with the gradient strips. While values overlapped for tetracyclines and fluoroquinolones (except levofloxacin), ECOFFs were lower than the ECOFFs for all macrolides and, conversely, higher for levofloxacin and rifampicin. Discrepancies have been reported in another study from Portugal [22]. Universal ECOFF values have not been formally assigned making difficult to ascertain wild-type and resistant field strains, and more data are needed for reaching a much-needed international standardization [7].

While we observed relatively concordant MIC results between BMD and LASARUS for fluoroquinolones, rifampicin, and amoxicillin/clavulanic acid, the BMD results were more concordant with the gradient strip results for macrolides (except for clarithromycin) and tetracyclines. Compared to the other two methods and similarly to a previous study [17], the gradient strip returned more elevated MIC values for both fluoroquinolones and rifampicin. This result can be partially explained by the known chelating effect of activated charcoal in the BCYE medium used in the Gradient strips. The degree of antimicrobial compound adsorption in a charcoal-containing medium can increase the MIC values, as reported by various studies [33–35]. This phenomenon is not expected in the LASARUS. However, we also reported significantly lower MIC values for amoxicillin/clavulanic acid using the gradient strip compared to the other two methods. Differences between antibiotics in the degree of their absorption and bioavailability in different media cannot be excluded. In order to surpass the known constraints of the gradient strip and the time-consuming and logistically difficult BMD approaches, the LASARUS medium offers some interesting advantages [17]: it is charcoal-free, and it allows inoculation of multiple samples using a multipoint inoculator. Additionally, it is a translucent medium allowing an easier and safer reading of the results (which could also be automated using optical readers). The gradient strips are not compatible with the current formulation of the LASARUS medium [17], so a combination of the two approaches is currently not possible. As shown already by another study [17], and confirmed by our data, overall the LASARUS medium looks as a promising alternative to BMD.

This study had some limitations. Only fraction of the total archived isolates at the National Reference Laboratory were tested. We tested mostly clinical samples from LD patients, so the resulting picture may not be fully representative of *L. pneumophila* bacteria circulating in Portugal (particularly in the environment). Information about antibiotic treatment was available for only around 20% of the isolates, and we cannot infer on the impact of potential antibiotic treatments administered to patients on the results of our assays. However, for the isolates for which the information was available, we did not observe any correlation between the type of treatment and the presence of reduced susceptibility to the corresponding compound.

Our results highlight the need for more extensive AST data on *L. pneumophila* in Portugal. Given the severity and potentially fatal outcome of LD, it is important to monitor the antimicrobial susceptibility status of circulating bacterial strains to identify the emergence of resistance in a timely manner. Whole genome sequencing of isolates with reduced antibiotic susceptibility will also be of great help to elucidate potential molecular determinants affecting the phenotype and giving rise to resistant strains.

### Supplementary Information

Below is the link to the electronic supplementary material.

Supplementary file1 (XLSX 1530 KB)

## Data Availability

The dataset generated during the current study is available as an Excel file in the Zenodo repository (10.5281/zenodo.8367288).
